# Distribution of Tetrodotoxin in Wild Pufferfish from the Southeastern Coastal Regions of China

**DOI:** 10.3390/toxins18100458

**Published:** 2026-10-20

**Authors:** Xinxin Zhang, Min Xu, Bei Chen, Shuilin Cai, Qilin Huang, Kun Qiao, Zhiyu Liu

**Affiliations:** 1 Key Laboratory of Cultivation and High-Value Utilization of Marine Organisms in Fujian Province, National Research and Development Center for Marine Fish Processing, Fisheries Research Institute of Fujian, Xiamen 361021, China; xinxinzhang5927@163.com (X.Z.);; 2 College of Food Science and Technology, Huazhong Agricultural University, Wuhan 430070, China

**Keywords:** tetrodotoxin, wild pufferfish, southeastern coast of China, ELISA

## Abstract

Wild pufferfish are abundant in the coastal waters of southeastern China, yet systematic data on their toxicity remain limited. This study collected 1230 specimens from Fujian, Guangdong, Guangxi, and Hainan and quantified tetrodotoxin (TTX) in nine tissues (eyes, skin, muscle, ovaries/testis, liver, digestive tract, kidneys, and heart) by ELISA. In total, 10 species were identified (*Lagocephalus wheeleri*, *L. gloveri*, *L. inermis*, *L. lunaris*, *Takifugu xanthopterus*, *T. ocellatus*, *T. niphobles*, *T. poecilonotus*, *T. bimaculatus*, *T. oblongus*). All tissues of *L. wheeleri* and *L. gloveri* contained TTX below the safety limit (2.2 μg/g), whereas the remaining eight species showed varying degrees of toxicity. *T. bimaculatus* and *T. oblongus* exhibited lethal-grade toxicity, with ovarian TTX reaching 320 μg/g and 393.56 μg/g, respectively. TTX was predominantly concentrated in the ovaries, liver, skin, and digestive tract, with substantial inter-individual variation. Marked sex-dependent differences and seasonal fluctuations were observed, driven by reproductive activity, water temperature, and prey availability. This study is the first to report that the wild *T. bimaculatus* from this region is a highly toxic species, and confirms the lethal toxicity of *T. oblongus*, providing a scientific basis for managing the consumption-related risks of wild pufferfish.

## 1. Introduction

Wild pufferfish poisoning represents a significant food safety and public health concern worldwide. Tetrodotoxin (TTX), one of the most potent non-protein neurotoxins known, is a weakly basic, colorless compound. Its molecular structure features a guanidinium group and a highly oxidized carbon skeleton containing a 2,4-dioxaadamantane domain and five hydroxyl groups [1]. TTX is physicochemically stable and heat-resistant; conventional cooking cannot effectively eliminate its toxicity [2,3], and no specific antidote is currently available [4]. TTX exerts its toxicity by specifically blocking voltage-gated sodium channels (Nav), thereby inhibiting action potential generation and nerve impulse conduction, which leads to the suppression of neuronal excitability, muscle paralysis, and acute poisoning symptoms. In mild cases, ingestion of TTX-contaminated food causes numbness of the lips and extremities, nausea, and vomiting. Severe cases may result in death due to respiratory muscle paralysis and heart failure [5,6]. TTX poisoning incidents associated with the consumption of wild pufferfish have been reported in many countries worldwide, posing a serious threat to public health [7,8,9].

China possesses abundant pufferfish resources, with over one hundred species documented, including more than 50 species within the Tetraodontidae family and 16 species belonging to the *Tetraodon* genus distributed along its coastlines [10]. In the southeastern coastal regions of China, pufferfish are frequently caught as bycatch during longline fishing and bottom trawling operations, while some edible pufferfish species are exported to markets such as Japan and South Korea. Moreover, China has a longstanding cultural tradition of consuming pufferfish, reflected in the folk saying “daring to eat pufferfish at the risk of one’s life.” Due to the high morphological similarity among wild pufferfish species, accurate species identification is challenging, and inadvertent consumption resulting from mixed catches can easily lead to poisoning.

A national surveillance study covering 2011–2020 [11] reported 92 pufferfish poisoning incidents, leading to 339 cases and 15 deaths (4.4% fatality rate). These cases were clustered along the southeast coast, mainly in Guangdong, Fujian, and Hainan, and peaked in March–April, coinciding with the pufferfish reproductive season when ovarian TTX rises. Fresh pufferfish was the leading source of poisoning, and risky dietary behaviors and improper home cooking (e.g., retaining liver) increased poisoning risk (RR = 1.8, 95% CI: 1.1–3.1). Despite public awareness of toxicity and consumption bans, wild pufferfish poisoning events still occur in coastal regions, including severe intoxication requiring ICU care [12]. However, information on species composition and toxicity of wild pufferfish in southeast coastal waters is limited, which warrants further investigation for food safety protection.

Existing studies have shown that TTX is one of the most toxic marine neurotoxins known to date. The oral LD_50_ in mice ranges from 232 to 532 μg/kg, and an intravenous LD_50_ is as low as 8 μg/kg [13,14,15]. Its toxicity is several hundred times greater than that of sodium cyanide [16,17]. The current detection methods for TTX can be categorized into the following types: mouse bioassays, cell-based assays, immunoassays, aptamer-based methods, biosensors, and instrumental analysis techniques. Among these, immunoassays rely on antigen–antibody-specific interactions and mainly encompass enzyme-linked immunosorbent assay (ELISA) and colloidal gold immunochromatographic test strips [5,18].

ELISA is ideal for the preliminary screening of large sample batches, with a detection sensitivity of 1 ng/mL [19]. Magnetic bead-based colorimetric immunoassays simplify operation and enable rapid TTX detection in shellfish, while colloidal gold test strips allow convenient on-site screening due to their portability and intuitive colorimetric readouts [5,20]. Biosensors combine biological recognition components with physicochemical transducers for signal amplification and efficient TTX detection, including electrochemical, SPR, SERS, and colorimetric sensors. Notably, a nanoenzyme-catalyzed microfluidic immunosensor can detect TTX within 10 min with a detection limit of 0.2 ng/mL, and multimodal sensors further enhance detection accuracy via signal cross-validation [21]. Instrumental analysis, particularly LC-MS/MS, serves as the gold standard for confirmatory TTX detection. It exhibits excellent selectivity and sensitivity, enabling precise quantification and structural identification of TTX and its analog, with a detection limit as low as 0.01 mg/kg [22]. Overall, different TTX detection methods vary significantly in sensitivity, throughput, portability, and accuracy. Given the large sample size and wide geographical coverage of this investigation on TTX distribution in pufferfish from southeast coastal waters, ELISA was selected as the primary detection method due to its simple operation, high throughput, and low cost.

Systematic data on the toxicity of wild pufferfish in the coastal waters of Fujian, Guangdong, Guangxi, and Hainan remain limited. This study presents the first large-scale quantitative survey of TTX in this region, covering 10 wild pufferfish species and nine tissues. ELISA was used to measure TTX levels in the eyes, skin, muscle, ovaries, testis, liver, digestive tract, kidneys, and heart. Toxin distribution and its seasonal dynamics were analyzed with respect to species, sex, tissue, and season. The aim was to systematically assess the food safety risks associated with wild pufferfish in this area and to provide a scientific basis for preventing accidental poisoning and managing TTX-related health risks.

## 2. Results and Discussion

### 2.1. Geographic Distribution of Wild Pufferfish

**Figure 1:**
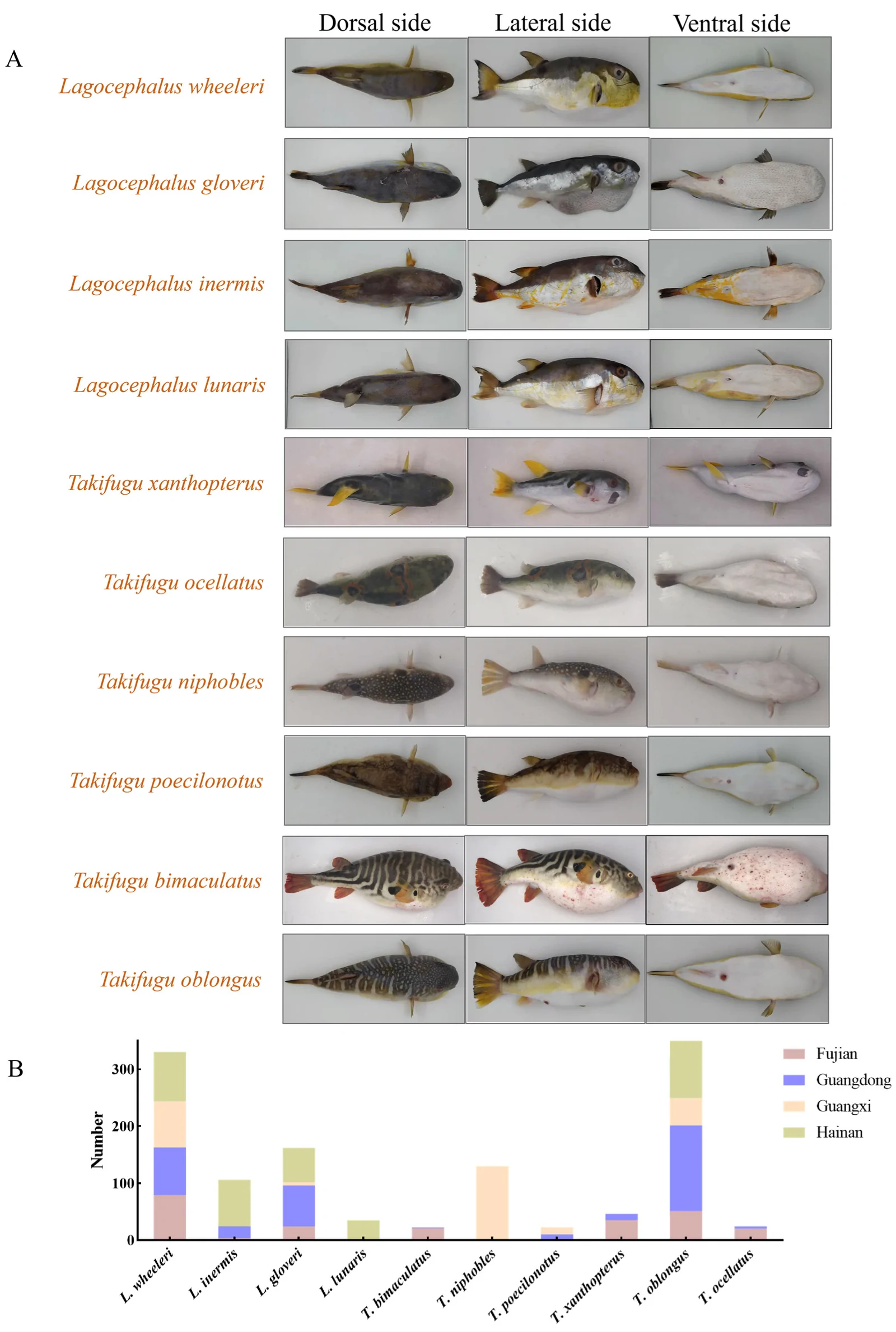
Morphology and catch quantities of the 10 wild pufferfish species. (**A**) Appearance of the 10 species (lateral, dorsal, and ventral views); (**B**) catch quantities of each species in the four provinces.

In this study, species identification was conducted on 1230 wild pufferfish specimens collected from the southeastern coastal waters of China. Based on morphological characteristics (short, blunt snouts resembling a rabbit’s nose; elongated, fusiform bodies; and either mostly smooth, spineless skin, as in *Lagocephalus*, or small spines densely covering the body and distinctive color patterns, as in *Takifugu)*, the specimens were assigned to ten species of the genera *Lagocephalus* and *Takifugu* (Table S1, Figure 1A) [23,24].

Coastal waters of southeast China support abundant wild pufferfish resources, although the distribution of toxicity is highly complex. Surveys from the Fujian and Guangdong coasts indicate that local pufferfish mainly belong to the genera *Lagocephalus* and *Takifugu*, with *Lagocephalus* comprising 75.8% of specimens [25]. Some representative species have well-documented distribution ranges: *L. spadiceus* is predominantly found in the South China Sea [26], while *L. gloveri* inhabits coastal waters of Changle and Guangdong [27]. *T. oblongus* occurs widely along the coasts of Hainan Island, Guangdong, Guangxi, Fujian, and Taiwan and can migrate into estuarine brackish waters [28].

Our survey (Figure 1B) further demonstrated that both *Lagocephalus* and *Takifugu* occurred across multiple provincial coastal waters of southeast China, with marked regional differences in species and abundance. Within *Lagocephalus*, *L. wheeleri* showed the widest distribution; we recorded 79, 84, 80, and 87 individuals in Fujian, Guangdong, Guangxi, and Hainan, respectively, with the largest catch from Hainan. *L. gloveri* was mainly collected in Hainan (82 individuals) and Guangdong (21 individuals), whereas *L. inermis* was more abundant in Guangdong (72 individuals) and Hainan (60 individuals). *L. lunaris* was only detected in Hainan (35 individuals). Within the genus *Takifugu*, *T. oblongus* was the most abundant species, with 150 individuals from Guangdong, 108 from Hainan, 51 from Fujian, and 48 from Guangxi. *T. niphobles* was exclusively captured in Guangxi (130 individuals). *T. poecilonotus* was mainly distributed in Guangdong and Guangxi, while *T. xanthopterus*, *T. ocellatus*, and *T. bimaculatus* were predominantly found along Fujian and Guangdong coasts.

### 2.2. Interspecific Toxicity Distribution of Wild Pufferfish

**Figure 2:**
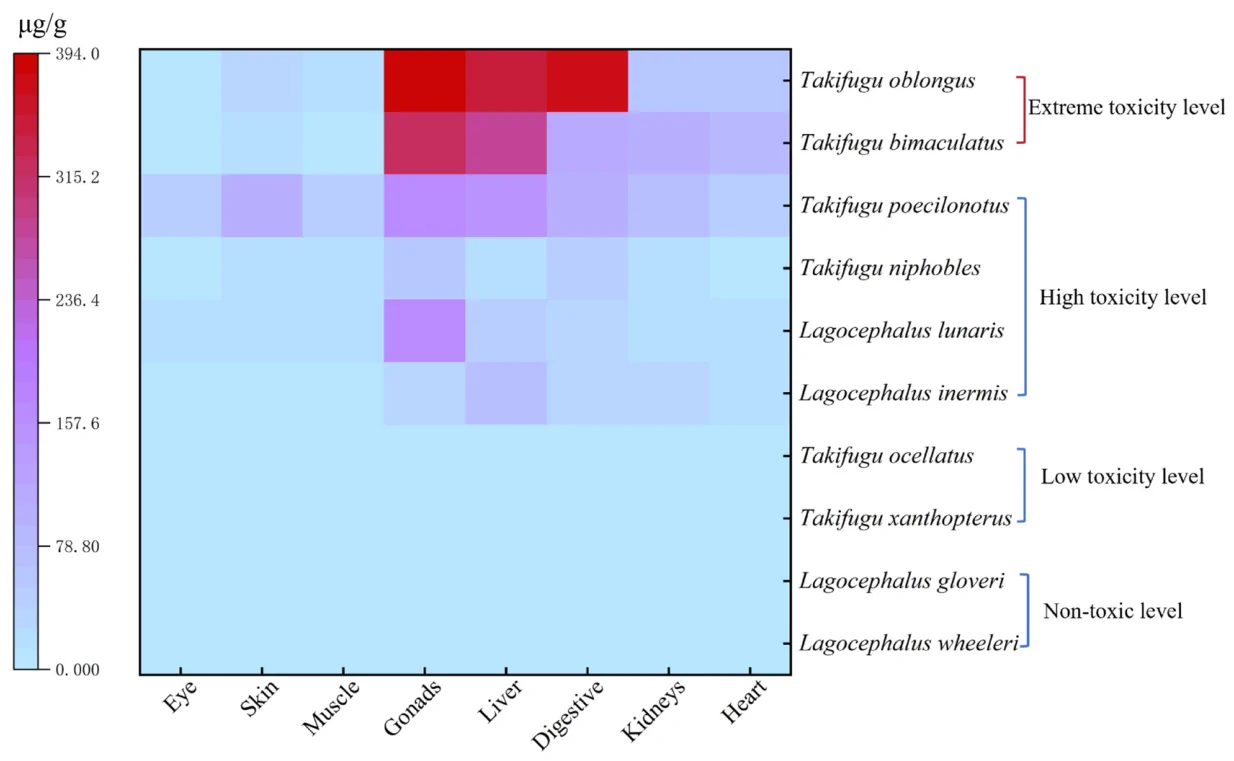
Toxicity classification of the 10 wild pufferfish species.

For pufferfish food safety assessment, worst-case scenarios should be prioritized for public health protection. Substantial inter-individual variability in TTX concentrations suggests that mean values may underestimate poisoning risks from highly toxic specimens; hence, maximum TTX concentrations were applied for conservative risk evaluation to determine the highest potential consumer exposure. Our interspecies comparison was designed to assess the maximum toxic potential of wild pufferfish species and identify the occurrence of highly toxic individuals, rather than quantify population-averaged toxicity. Toxicity levels and rankings for each tissue were visualized in a heatmap (Figure 2). Most species showed high toxicity; *T. bimaculatus* and *T. oblongus* reached the lethal toxicity grade (maximum TTX: 320 μg/g and 393.56 μg/g, respectively), whereas *L. wheeleri* and *L. gloveri* were classified as non-toxic (maximum TTX: 1.79 μg/g and 1.27 μg/g). Toxicity differed significantly among species. Even among species collected from the same waters of Fujian, such as *L. wheeleri*, *T. xanthopterus* (maximum TTX: 4.87 μg/g) and *T. bimaculatus*, toxicity differed markedly.

Consistent with the TTX bioaccumulation hypothesis, Hibi et al. [29] analyzed the intestinal contents of wild toxic and non-toxic *T. rubripes* and found that toxic individuals consumed significantly more TTX-bearing benthic organisms, while non-toxic individuals mainly preyed on fish. In addition, studies have demonstrated that pufferfish can efficiently bioaccumulate TTX by ingesting eggs from other toxic pufferfish [30]. Sympatric pufferfish species such as *Sphoeroides spengleri* and *S. testudineus* primarily feed on crustaceans and mollusks but exhibit distinct prey preferences [31]. Most fish can detect low TTX concentrations via gustation and reject contaminated prey [32], whereas pufferfish perceive TTX through olfaction [33,34]. This specialized sensory and feeding trait facilitates TTX accumulation in pufferfish.

Against this literature background, we compared the feeding habits of three pufferfish species in the same sea area. All three species consume mollusks, crustaceans, and small fishes yet show clear divergence in feeding preferences. *L. wheeleri* feeds primarily on mollusks and crustaceans with fishes as secondary prey; only qualitative dietary records exist for this species, with no quantitative stomach-content data [35]. *T. xanthopterus* possesses a wider trophic spectrum, in which small fishes dominate its diet and cannibalism is observed. It also preys on cephalopods, crustaceans, and bivalves, with mobile prey representing a major dietary component [36]. *T. bimaculatus* relies predominantly on benthic crustaceans and shellfish, with small fishes as minor supplementary prey, and cannibalism is rare [37]. On the basis of these interspecific feeding differences, we hypothesize that dietary divergence constitutes an important driver underlying the marked interspecific variation in TTX accumulation, with important implications for food safety risk assessment of pufferfish products.

### 2.3. Tissue-Specific Distribution of TTX in Wild Pufferfish

**Figure 3:**
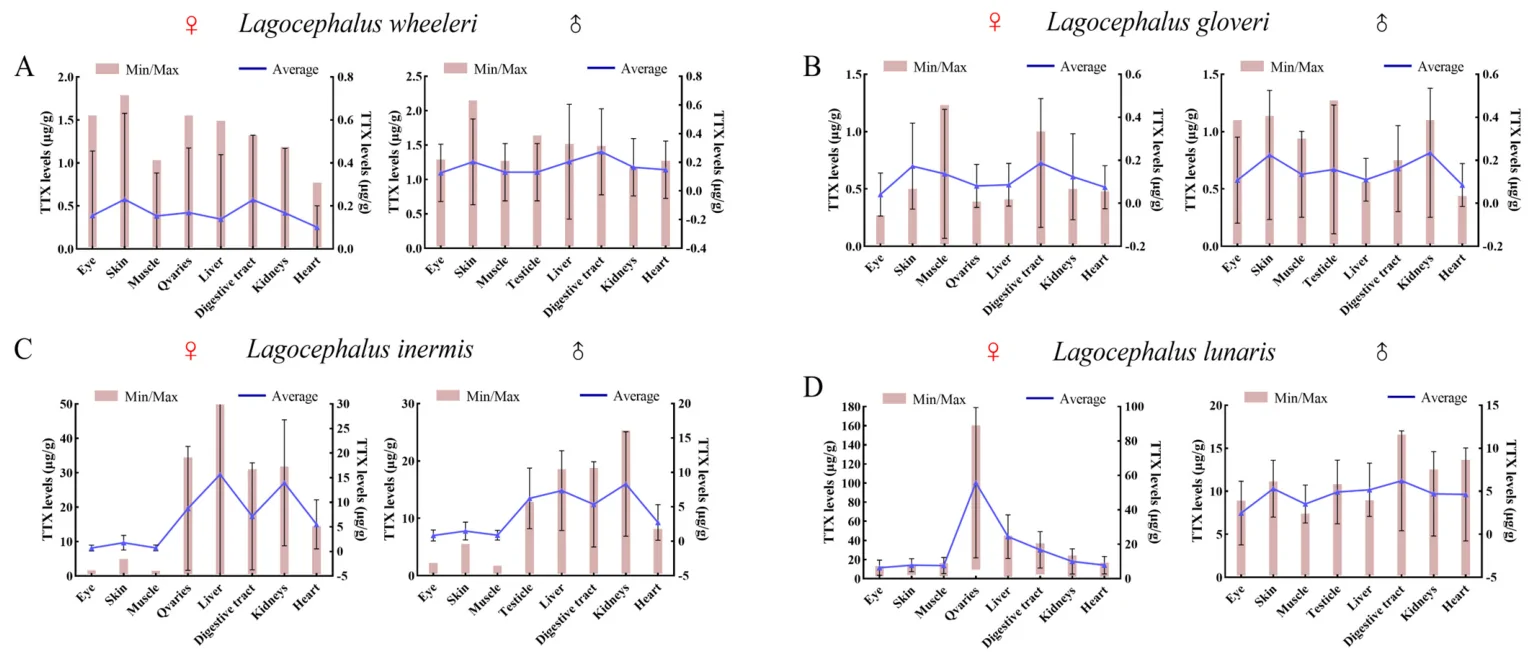
TTX levels in various tissues of four *Lagocephalus* species. (**A**) *L. wheeleri*; (**B**) *L. gloveri*; (**C**) *L. inermis*; (**D**) *L. lunaris*. Data are presented as the mean and the maximum–minimum range. TTX: tetrodotoxin; ♀: female; ♂: male.

**Figure 4:**
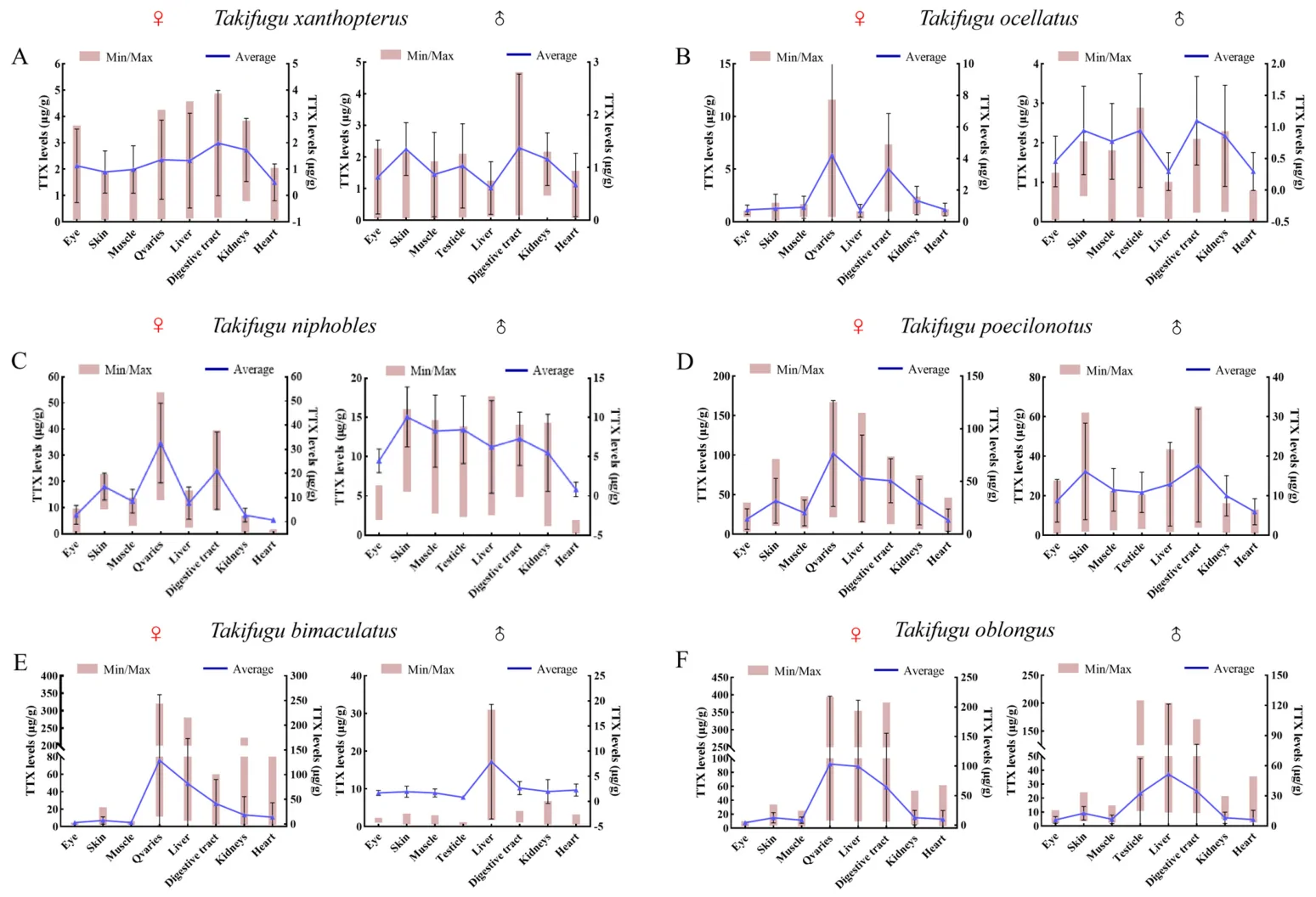
TTX levels in various tissues of six *Takifugu* species. (**A**) *T. xanthopterus*; (**B**) *T. ocellatus*; (**C**) *T. niphobles*; (**D**) *T. poecilonotus*; (**E**) *T. bimaculatus*; (**F**) *T. oblongus*. Data are presented as the mean and the maximum–minimum range. TTX: tetrodotoxin; ♀: female; ♂: male.

TTX concentrations across tissues from the 10 wild pufferfish species investigated in this study are presented in Figure 3 and Figure 4, with detailed numerical data summarized in Table S2. Overall, our results revealed marked interspecific and tissue-specific variation in TTX bioaccumulation. Consistent with prior reports [38,39], the ovary, liver, digestive tract, and skin generally served as major TTX-accumulating tissues, while muscle tissues typically exhibited lower toxin concentration. Nevertheless, our study further documented widespread TTX occurrence across nearly all examined tissues. Although TTX concentrations in eyes and muscle were generally lower than those in ovaries, livers, and digestive tracts, relatively high TTX levels were detected in these conventionally low-risk tissues for certain species. For instance, female *T. poecilonotus* and *T. oblongus* contained up to 39.8 μg/g and 10.12 μg/g TTX in eyes, and up to 48 μg/g and 24 μg/g TTX in muscle, respectively, which raises notable food safety concerns.

Within the genus *Lagocephalus*, our data showed uniformly low TTX concentrations across all tissues of *L. wheeleri* and *L. gloveri*, classifying these two species as non-toxic. In contrast, *L. inermis* and *L. lunaris* displayed pronounced tissue-dependent TTX patterns. For *L. inermis*, the liver (maximum 71.55 μg/g) and ovary (maximum 34.42 μg/g) represented the most toxic tissues, whereas muscle and eye tissues were non-toxic, with peak TTX detected in the kidney. *L. lunaris* was the most toxic species among the four *Lagocephalus* species examined in this study, with high TTX levels observed in the ovary, liver, digestive tract, and kidney. For *Takifugu*, our observations agreed with the established tissue distribution pattern of TTX in this genus [17,40]. In the present study, *T. niphobles*, *T. ocellatus*, *T. bimaculatus*, and *T. oblongus* all had highly or extremely toxic ovaries (e.g., up to 393.56 μg/g in *T. oblongus*), and peak TTX concentrations were found in the liver for *T. bimaculatus* and *T. oblongus*.

Across both sexes, liver, digestive tract, and skin were highly enriched in TTX, and ovaries accumulated the highest toxin during reproductive periods. Earlier feeding experiments indicated that the liver acts as the primary organ for initial TTX uptake after oral ingestion: hepatic TTX concentrations rise rapidly upon exposure and subsequently decline as TTX is translocated to other target storage tissues [41]. Our interspecies tissue profiles support this model, reinforcing the liver’s role as a TTX transit hub. Previous work also demonstrated developmental differences in TTX handling: at identical TTX doses, juvenile pufferfish initially sequester TTX in the skin prior to excretion, while adults steadily accumulate TTX in the liver [42]. The skin-stored TTX is widely considered an anti-predator defense. Its accumulation relies on specialized structures such as TTX secretory glands and cystic cells rather than passive diffusion [43], and TTX present in juvenile mucus can deter predators [44]. Our tissue data further corroborate that the skin participates in dynamic TTX trafficking, with its toxin burden regulated by hepatic transport processes [45].

The digestive tract was also among the TTX-enriched tissues in the species described above; in *T. oblongus*, the maximum concentration in the digestive tract reached 378 μg/g, which is classified as lethal toxic. This finding supports the hypothesis that TTX accumulates in pufferfish through the food chain: TTX is initially produced by marine bacteria and is subsequently transferred to pufferfish via toxic prey such as flatworms, snails, and crustaceans [17,46]. Crustaceans, such as crabs and gastropods, as well as mollusks, are potential vectors for TTX transmission through the food chain. A survey of large benthic organisms in the intertidal zones of Fujian’s islands identified a total of 862 species, including 248 mollusk species (28.77%), 181 crustacean species (21.0%), and 163 polychaete species [47]. The *Acinetobacter* sp. *P1332* strain, a tetrodotoxin-producing bacterium, was isolated by Liang et al. [48] from the liver of *T. oblongus* collected off the Fujian coast in the East China Sea. Although reports of TTX-producing bacterial genera in the waters off China’s southeastern coast are limited, the temperature ranges of toxin-producing bacteria isolated from neighboring waters (Japan and Vietnam) suggest that southeastern coastal waters of China provide ecological conditions favorable to TTX-producing bacteria [49,50].

### 2.4. Sexual Distribution of TTX in Wild Pufferfish

**Figure 5:**
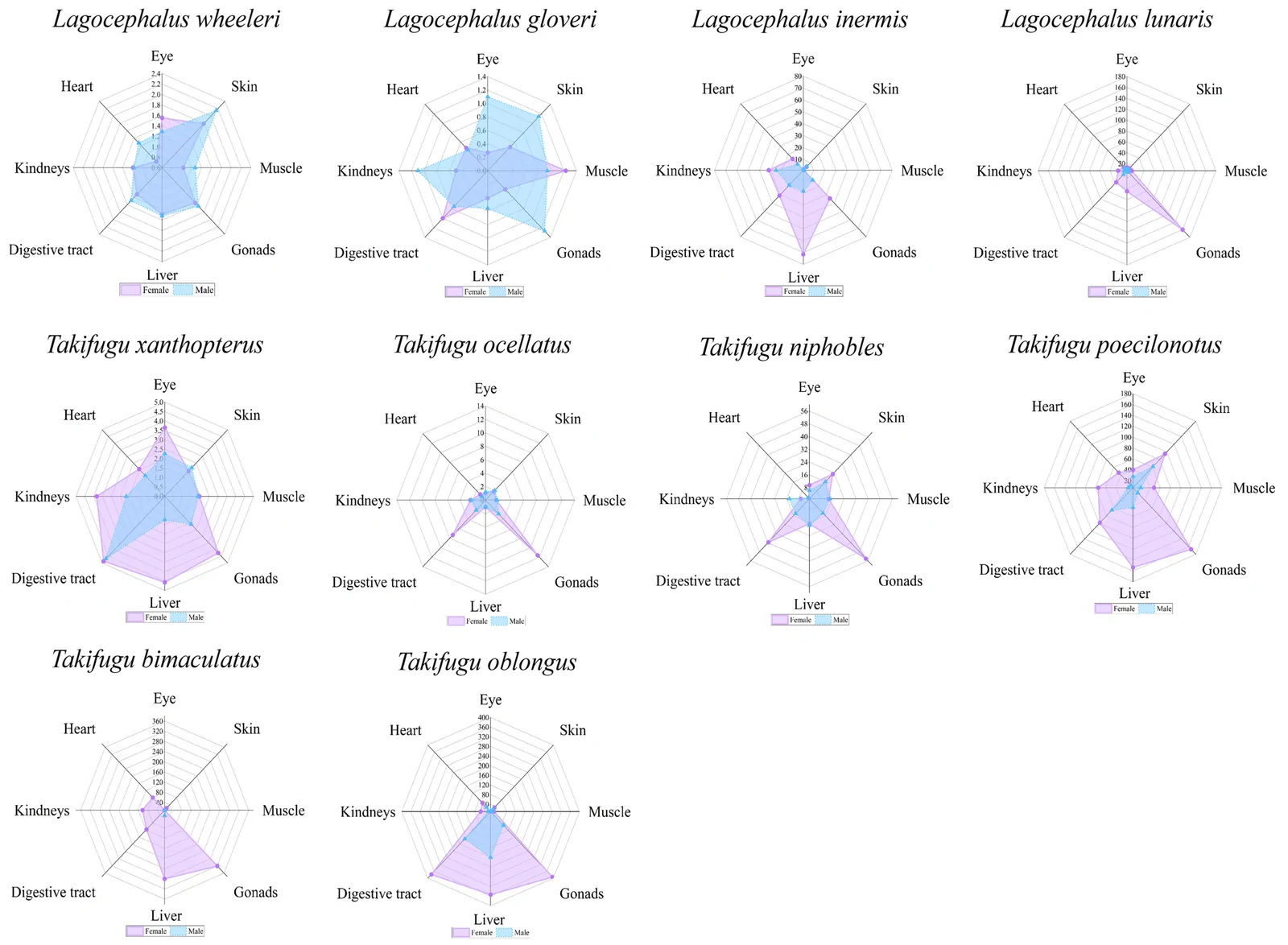
Differences in TTX distribution between male and female tissues of the 10 wild pufferfish species.

TTX distribution showed significant sex-dependent differences. In toxic pufferfish, females mainly accumulate TTX in the ovaries, liver, digestive tract, and skin, while males mainly accumulate it in the liver, digestive tract, and skin. Overall, TTX levels were higher in females than in males (Figure 5). No sex differences in toxicity were detected in *L. wheeleri* or *L. gloveri*. TTX levels in female *L. inermis* were significantly higher than those in males, especially in the gonads (female [F] 34.42 vs. male [M] 12.82 μg/g), liver (F 71.55 vs. M 18.57 μg/g), and digestive tract (F 31.06 vs. M 18.79 μg/g). In *L. lunaris*, sex differences were pronounced in the kidneys (F 24.32 vs. M 12.54 μg/g), digestive tract (F 37 vs. M 16.56 μg/g), liver (F 44.9 vs. M 8.86 μg/g), and gonads (F 160.26 vs. M 10.81 μg/g), with toxicity significantly higher than in males.

Within the genus *Takifugu*, female *T. xanthopterus* generally exhibited weakly toxic or non-toxic tissues. Although TTX levels in tissues other than the skin were higher in females than in males, these differences did not reach the threshold for toxicity level differentiation. In female *T. ocellatus*, TTX was significantly concentrated in the digestive tract (F: 7.73 vs. M: 2.1 μg/g) and gonads (F: 11.6 vs. M: 2.89 μg/g); other tissues showed no significant sex differences. Similarly, in female *T. niphobles*, the skin (F 22.94 vs. M 16.08 μg/g), digestive tract (F 39.5 vs. M 14.05 μg/g), and gonads (F 54 vs. M 13.85 μg/g) showed higher toxicity, whereas TTX levels in other tissues were similar to those in males. All tissues in female *T. poecilonotus* were highly toxic, with significant sex differences across nearly all tissues except the skin (F 95 vs. M 62 μg/g), digestive tract (F 98 vs. M 65.05 μg/g), and liver (F 153.51 vs. M 43.48 μg/g). In *T. bimaculatus* and *T. oblongus*, sex differences were particularly pronounced in the gonads, liver, and digestive gland. In *T. bimaculatus*, values were gonads (F 320 vs. M 1.13 μg/g), liver (F 280 vs. M 31 μg/g), and digestive gland (F 117 vs. M 4.11 μg/g); in *T. oblongus*, the corresponding values were gonads (F 393.36 vs. M 90 μg/g), liver (F 393.36 vs. M 90 μg/g), and digestive gland (F 393.36 vs. M 90 μg/g). The sex-dependent differences in TTX levels described above likely arise from the dual ecological roles of TTX in chemical defense and reproduction, which impose distinct selective pressures on females and males. In females, a substantial proportion of accumulated TTX is allocated to the ovaries and subsequently transferred to the eggs, protecting both eggs and larvae [45,51]. This reproductive allocation directly explains why females often carry higher TTX levels than males, particularly in reproductively active individuals.

This allocation is developmentally and hormonally regulated. In *T. pardalis*, the skin acts as the main TTX storage organ during early ovarian development, but once the ovaries reach the yolk-globule stage, they take over as the dominant accumulation site, holding 53–76% of total body toxin [51]. Female TTX accumulation is, therefore, not static but shifts toward the gonads as reproduction approaches, a pattern not seen in males. Consistent with this, ovarian TTX and its analogs levels correlate positively with *β*-estradiol [52], pointing to direct modulation by sex-hormone pathways. Yin et al. [53] further identified an ovary-specific TTX-binding protein that sequesters TTX via TTX–vitellogenin complexes, again linking hormonal regulation to sex-biased accumulation. PSTBP, another TTX-binding protein, mediates TTX transport from blood to target organs and facilitates liver-to-ovary transfer during maturation [54,55,56]. This pathway helps explain the elevated ovarian TTX in mature females and supports the liver–ovary axis, rather than peripheral tissues alone, as the driver of female-biased TTX accumulation. In addition, TTX acts as a pheromone that attracts males to females [34,57]. This signaling role may reinforce sex differences in TTX distribution: females may gain an advantage by keeping TTX high in specific tissues as a chemical cue, whereas males may allocate TTX primarily for defense rather than for reproductive signaling or gamete protection.

### 2.5. Seasonal Differences in Toxicity Among Pufferfish Species

To examine how tissue toxicity varies throughout fish breeding and maturation cycles within a year, seasons were defined according to photoperiod (day length), as the sampling sites are located in tropical and subtropical monsoon climate zones. Accordingly, the year was divided into spring (March–May), summer (June–August), autumn (September–November), and winter (December–February) [58]. Samples of *T. bimaculatus* and *T. niphobles* were collected in only a single season, precluding seasonal comparison. Except for *L. wheeleri*, all species lacked complete sampling across the four seasons, mainly due to fishing moratoriums and field sampling constraints.

**Figure 6:**
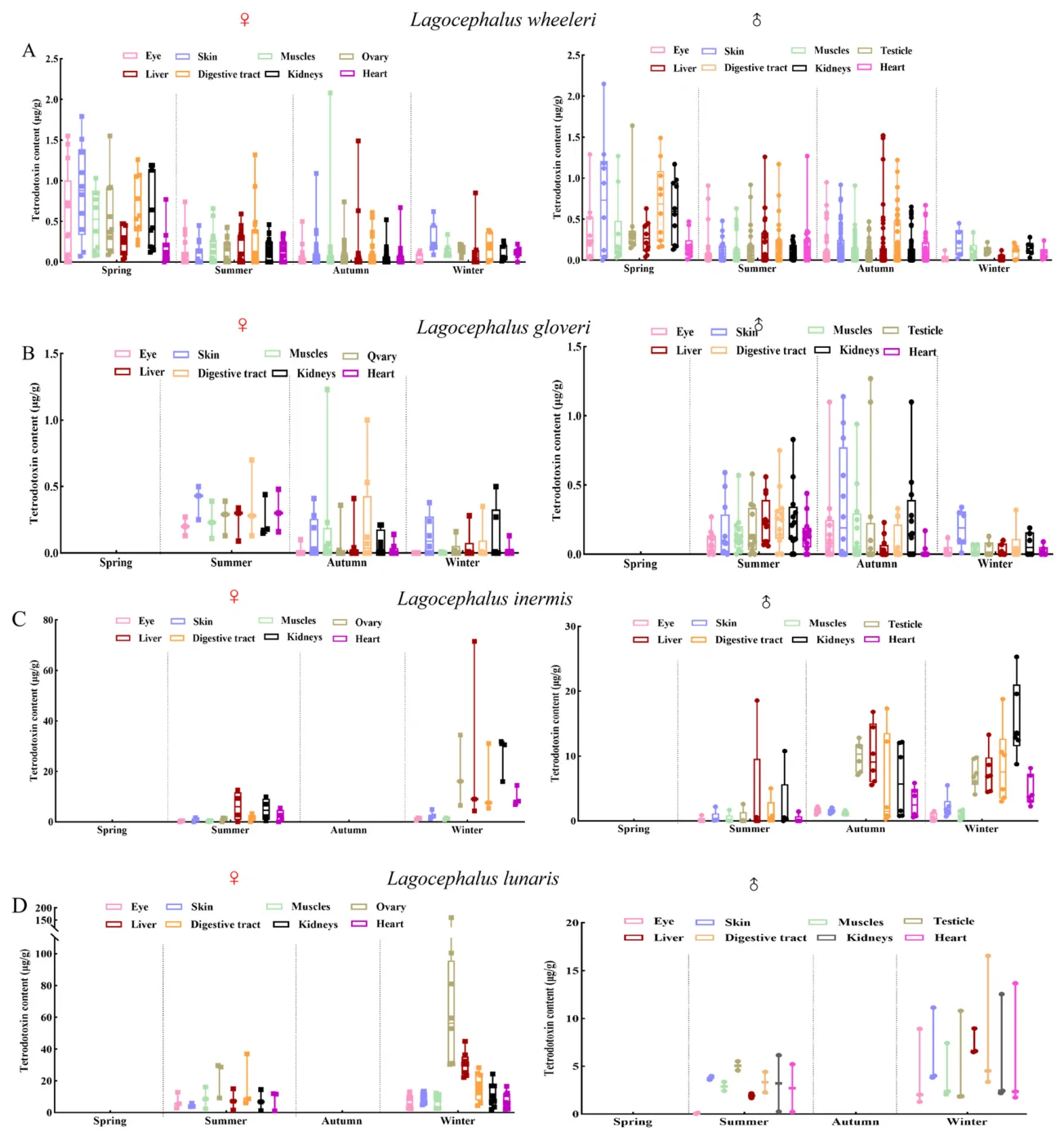
Seasonal dynamics of TTX levels in various tissues of the four *Lagocephalus* species. (**A**) *L. wheeleri*; (**B**) *L. gloveri*; (**C**) *L. inermis*; (**D**) *L. lunaris*.

**Figure 7:**
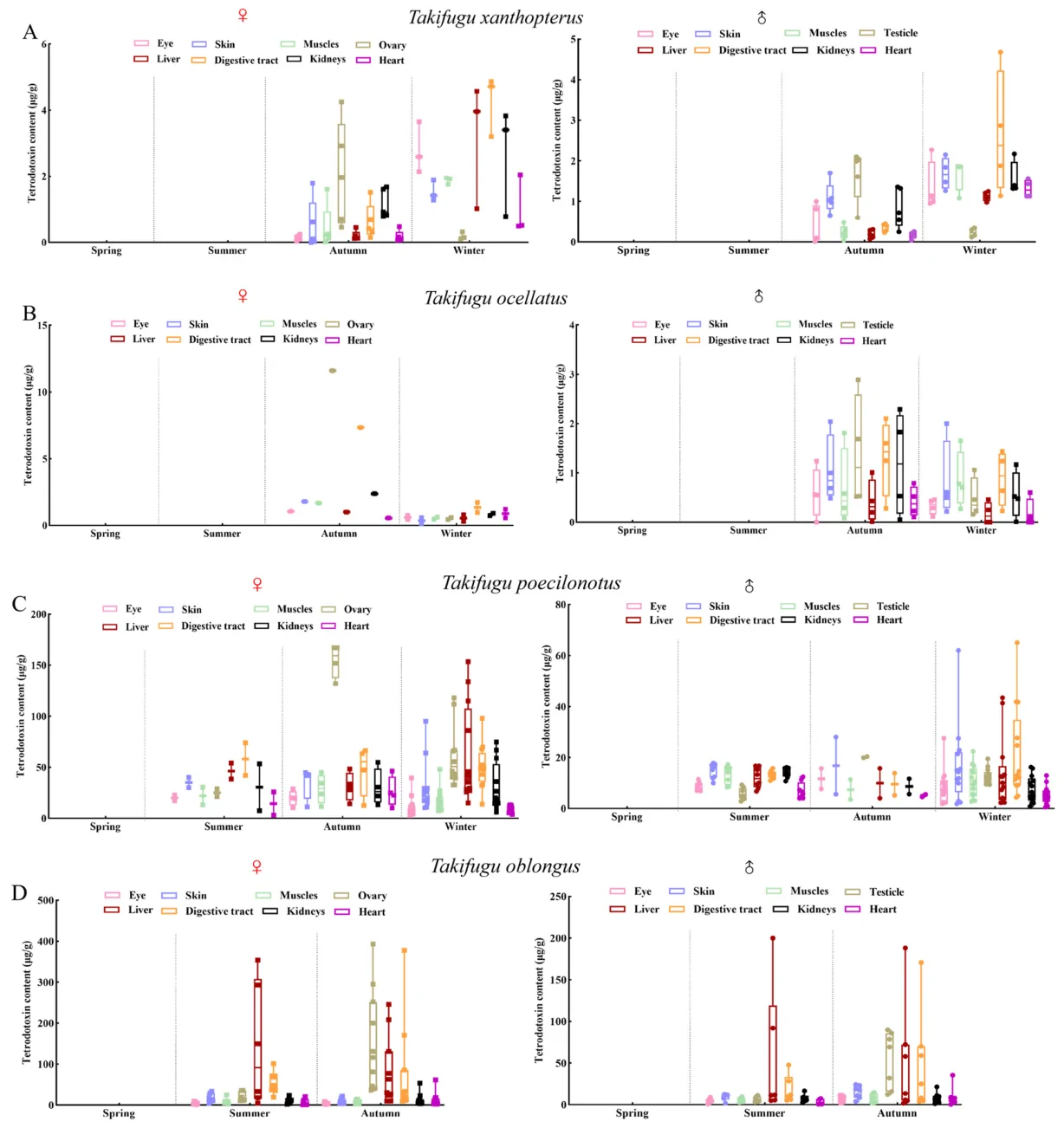
Seasonal dynamics of TTX levels in various tissues of the four *Takifugu* species. (**A**) *T. xanthopterus*; (**B**) *T. ocellatus*; (**C**) *T. poecilonotus*; (**D**) *T. oblongus*.

Seasonal variation in TTX content across tissues is illustrated in Figure 6 and Figure 7, and the statistical comparisons are summarized in Table S3. TTX levels in all tissues of *L. wheeleri* and *L. gloveri* remained below 2.2 μg/g throughout the year, consistent with their classification as non-toxic. This seasonal variation in TTX tissue distribution may be associated not only with the reproductive physiological demands of the spawning period, but also with temperature-driven fluctuations in TTX sources within the food chain, as well as tissue-specific toxin transport and hepatic metabolism. The maximum TTX concentrations in the tissues of *L. inermis* were one toxicity grade higher in winter than in summer. Populations of *L. inermis* in the southeastern Arabian Sea off India exhibited two spawning peaks: a major peak from November to February and a secondary peak in August [59]. The sexual difference was particularly pronounced in *L. lunaris*: ovarian TTX concentrations in females varied significantly with season (*p* < 0.05), reaching maximum values of 29.63 μg/g in summer and 160.26 μg/g in winter. By contrast, seasonal variation in TTX levels across tissues in males was relatively minor. These findings suggest that female pufferfish lose ovarian TTX after ovulation in summer and subsequently re-accumulate the toxin through intensive feeding in autumn and winter, leading to markedly elevated toxicity in winter [60]. *L. lunaris* generally reaches sexual maturity in November, a period when TTX is transported to and accumulated in the ovaries [50].

In female *T. xanthopterus*, TTX distribution differed significantly between autumn and winter in all tissues except the eyes, digestive tract, and kidneys (*p* < 0.05). Specifically, ovarian TTX was high in autumn but decreased in winter, whereas TTX in the liver increased. Males showed similar trends, with significant differences in the muscle, testis, liver, digestive tract, and heart (*p* < 0.05). Female *T. ocellatus* showed higher maximum TTX levels in autumn than in winter in all tissues. Female *T. poecilonotus* showed marked seasonal variation in TTX across tissues, with the highest ovarian TTX level recorded in autumn (167 μg/g). *T. oblongus* differed from the species described above: its maximum TTX level in the liver was significantly higher in summer than in autumn, whereas TTX levels in the ovary and testis were significantly higher in autumn than in summer (*p* < 0.01). Seasonal TTX variation is species-dependent. The autumn TTX peak in the aforementioned species may be attributed to reproductive cycles (TTX decreases after spawning and increases again via feeding), physiological modulation of toxin accumulation for overwintering defense, seasonal changes in TTX levels in prey, and water temperature variation affecting the physiology of pufferfish. Consistent with these proposed drivers, Varini et al. [16], in their review of relevant studies from 2017 to the present, found that TTX accumulation patterns exhibit seasonal fluctuations and are closely related to water temperature, corroborating temperature as a key modulator of seasonal TTX dynamics. Qian et al. [61] further reported that decreasing water temperature markedly affected the physiology of *T. obscurus*: at 11 °C, this species stopped feeding and its activity declined sharply. Such temperature-mediated alterations in feeding and activity offer a plausible mechanistic explanation for the association between water temperature and seasonal TTX accumulation in pufferfish.

Seasonal shifts in TTX-producing bacterial communities may influence TTX accumulation in pufferfish. Studies have found that among strains isolated under 25 °C culture conditions, *Vibrio* and *Pseudomonas* are the two dominant genera. However, most summer isolates can grow at 35 °C, whereas winter isolates cannot. This seasonal difference in growth temperature range directly reflects adaptive differentiation of the microbial community in response to water temperature [62]. A 2019 survey of *Vibrio* communities across four seasons in Dongshan Bay (southern Fujian) showed significant seasonal variation in *Vibrio* abundance: highest in summer (5.37 × 10^6^ copies/L), followed by autumn (4.58 × 10^6^), spring (1.18 × 10^6^), and winter (1.55 × 10^4^), with the summer–winter difference exceeding two orders of magnitude. Temperature, dissolved oxygen, nitrate, and nitrite are the primary environmental factors driving this variation [63]. However, seasonal variations in *Vibrio* abundance do not necessarily correspond to seasonal variations in the activity of TTX-producing bacteria. To date, no study has conducted a paired analysis of seasonal variations in TTX levels in pufferfish alongside the abundance of TTX-producing bacterial communities in the same waters; this warrants further investigation in the future.

A major limitation of this study is that TTX quantification relied on ELISA. Although this method is suitable for detecting immunoreactive TTX, it cannot differentiate between TTX and its analogs. Therefore, it is possible that some TTX is metabolized in the liver into analogs with limited or no cross-reactivity with the ELISA capture antibody, resulting in a negative quantification bias. Based on the available data, the TTX concentrations report ed in this study are likely lower than the actual TTX equivalents. Future studies should employ analytical techniques with higher selectivity, such as LC-MS/MS, to validate ELISA results, identify potential TTX analogs, and more accurately assess the impact of metabolic conversion on toxin distribution.

In summary, the southeastern coast of China lies in a monsoon climate zone in which seawater temperature, liver metabolism and prey communities vary seasonally; these factors may jointly influence TTX production, transfer, and bioaccumulation. Because sampling depended on fishing operations and was interrupted by seasonal fishing moratoriums, complete four-season datasets were not obtained for most species, so annual trends could not be fully resolved. Accordingly, the only firm conclusion that can be drawn is that TTX accumulation varies seasonally.

## 3. Conclusions

Through toxicity analysis of 10 wild pufferfish species from the southeastern waters of China, this study confirmed that *T. oblongus* and *T. bimaculatus* are highly toxic species, whereas TTX was not detected at toxic levels in *L. wheeleri* and *L. gloveri*. This study clarifies the toxicity profile of pufferfish in this region and provides direct evidence for distinguishing high-risk from low-risk species. Within species, TTX distribution showed clear tissue specificity and sex-dependent difference: during the breeding period, most females had higher TTX levels than males, and the toxin was concentrated mainly in the ovaries, liver, skin, and digestive tract. TTX content also varied significantly with season: in most females, ovarian TTX peaked during the breeding season, decreased after ovulation, and increased again with intensified feeding. Overall, this study characterizes the species-specific toxicity, tissue distribution, and temporal variation in wild pufferfish in the southeastern waters of China, providing a regional baseline of TTX data for future risk monitoring and species management.

## 4. Materials and Methods

### 4.1. Samples

A total of 1230 wild pufferfish specimens were collected from the coastal waters of Fujian, Guangdong, Guangxi, and Hainan provinces, China. The fish samples were obtained as incidental catch by fishing boats from September 2019 to October 2020. After collection, basic information such as the sampling location (sea area number), sampling month, and sample size was recorded on site (Table S4). The samples were then placed in an insulated box, stored on crushed ice for preservation, and transported to the laboratory as quickly as possible. During transportation and storage, the samples were maintained at low temperatures until analysis. During the seasonal difference analysis, the gonadosomatic index (GSI) combined with gonadal morphology was used to determine the sexual maturity of pufferfish specimens [64].

### 4.2. TTX Extraction

Pufferfish species were identified based on morphological characteristics. Body length and body weight were recorded for each specimen. After dissection, the eyes, skin, muscle, ovaries, testes, liver, kidneys, digestive tract, and heart were separated, weighed, and recorded. TTX was extracted according to the National Food Safety Standard for the Determination of Tetrodotoxin in Aquatic Products (GB 5009.206-2016) [65], with modifications. Briefly, 5 g each of skin, muscle, and liver and 1 g each of the other tissues were accurately weighed into a centrifuge tube (individual tissue samples with a mass below 1 g were pooled and subjected to combined detection). Four volumes of 0.1% (*v*/*v*) acetic acid solution (AR, Sinopharm, Shanghai, China) were added, and the mixture was homogenized and left to stand at 4 °C for 12 h. The mixture was then ultrasonicated for 10 min, centrifuged at 8000 rpm for 5 min, and the supernatant was collected. Four volumes of 0.1% acetic acid solution were added to the solid residue, and the homogenization and ultrasonication steps were repeated for 10 min. The mixture was then boiled in a hot water bath for 10 min. After cooling, it was centrifuged again at 8000 rpm for 5 min, and the two supernatants were combined. Finally, the pH was adjusted to 6.5–7.0 with 1 mol/L NaOH solution (AR, Sinopharm, Shanghai, China), and 0.01 M PBS solution (Biosharp, Hefei, China) was added to bring the total volume to ten times the weight of the residue. The resulting solution was used immediately for testing.

### 4.3. ELISA Quantification

**Figure 8:**
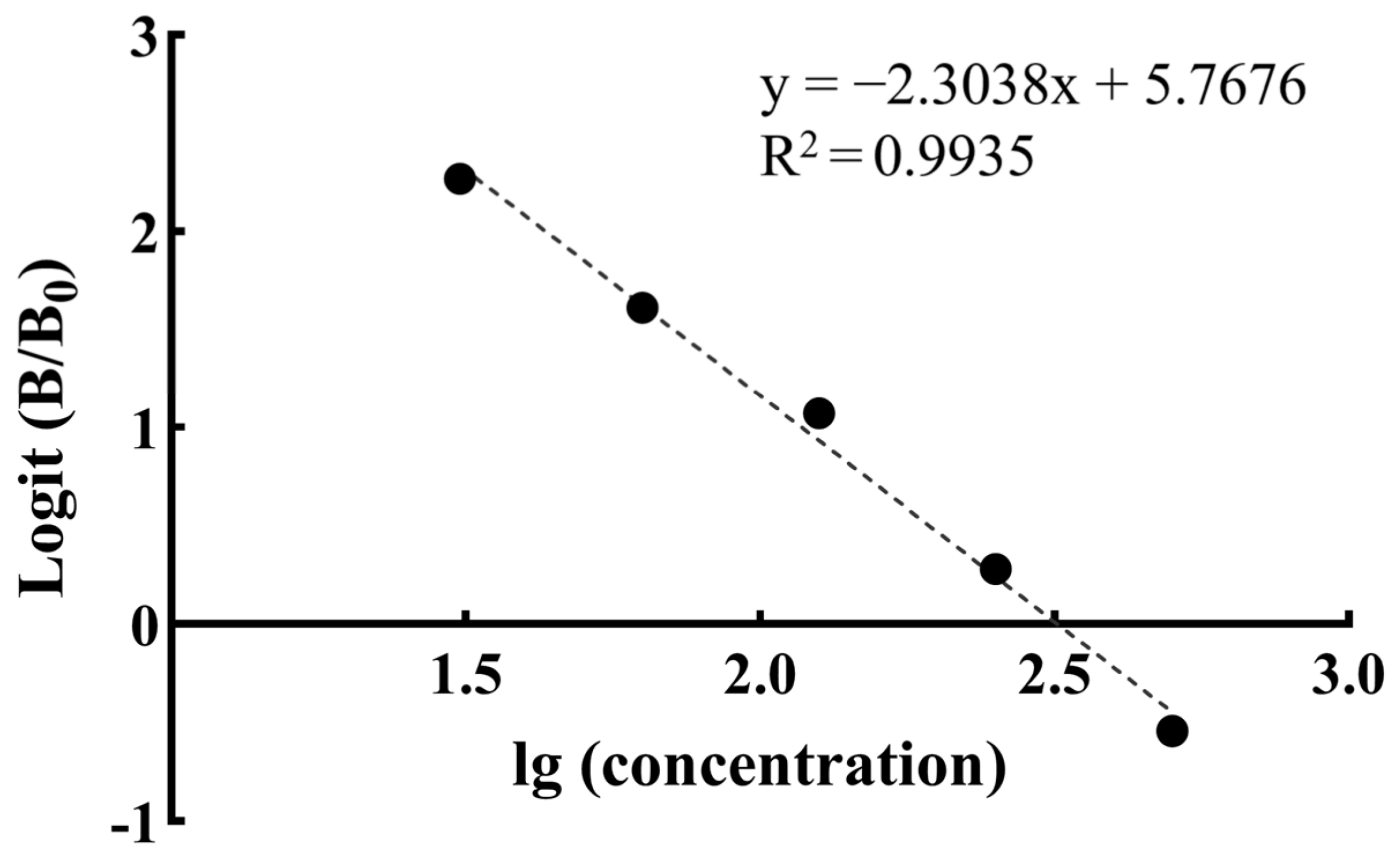
The logit–log calibration curve was fitted from 31.25 to 500 ng/mL. The limit of detection (LOD) was 0.07 ng/mL, with the regression equation y = −2.3038x + 5.7676 (R^2^ = 0.9935). The calculated IC_90_ and IC_80_ values were 35.48 ng/mL and 79.83 ng/mL, respectively. The IC_50_ was 318.83 ng/mL.

Indirect competitive ELISA was then used for the quantitative analysis of TTX. The ELISA procedure was as follows: plates were coated with the antigen (BSA-TTX) overnight at 4 °C, followed by blocking, air-drying, and storage at −20 °C. TTX standards and enzyme-labeled antibodies (anti-TTX mAb and HRP-goat anti-mouse lgG (Proteintech, Wuhan, China)) were added and incubated at 25 °C for 30 min. After washing, TMB substrate (PR1200, Solarbio, Beijing, China) was added for color development, and the reaction was terminated with sulfuric acid. Optical density was measured at 450 nm, and TTX content in the samples was quantified using a standard curve (Figure 8).

### 4.4. Toxicity Grading Criteria

According to the Fujian provincial local standard DB35/T 532-2004 [66] and the regulations of the Japanese Ministry of Health, Labour and Welfare [67], tissue TTX contents were graded as follows: non-toxic grade, TTX < 2.2 μg/g (corresponding to <10 MU/g); weakly toxic grade, 2.2 μg/g ≤ TTX < 22 μg/g; strongly toxic grade, 22 μg/g ≤ TTX < 220 μg/g; and lethal toxicity grade, TTX ≥ 220 μg/g.

### 4.5. Statistical Analysis

All data were statistically analyzed. Continuous variables were expressed as the mean ± standard deviation (mean ± SD). The Kruskal–Wallis test was used for multiple-group comparisons, and Dunn’s test was used for post hoc pairwise comparisons. The significance level was set at α = 0.05, and *p* < 0.05 was considered statistically significant. In addition, risk assessment and toxicity classification were performed based on the maximum TTX content in each tissue and the toxicity grading criteria.

## Data Availability

The original contributions presented in the study are included in the article/Supplementary Materials. Further inquiries can be directed to the corresponding author.

## Supplementary Materials

The following supporting information can be downloaded at: https://www.mdpi.com/article/10.3390/toxins18100458/s1. Table S1: External Morphology of 10 Pufferfish Species; Table S2: Summary of TTX data in different tissues detected by ELISA; Table S3: Seasonal Statistical Analysis; Table S4: Summary of sample information.
