# Supplementary material for: Distribution of Tetrodotoxin in Wild Pufferfish from the Southeastern Coastal Regions of China
Source: Toxins (Basel). 2026 Oct 20;18(10):458. doi: 10.3390/toxins18100458 (PMC13709651; doi:10.3390/toxins18100458)
Supplement: Supplementary file 1 [file toxins-18-00458-s001.zip › Table S1.pdf]

Table S1 External Morphology of 10 Pufferfish Species

Species	Body colouration and markings	Prickles	Fins characteristics
L. wheeleri	Lateral side with a light silvery longitudinal stripe	Head, dorsum, and venter covered with prickles; dorsal spinulation not extending to the dorsal-fin origin.	Caudal-fin lower margin with a broad grey edge.
L. gloveri	Lateral side with a dark silvery longitudinal stripe.	Lateral flanks smooth and scaleless; prickles restricted to head, dorsum, and venter.	Caudal fin doubly emarginate posteriorly; upper and lower lobes mostly white.
L. inermis	Dorsal half of body yellowish-brown; distinguished by a black gill membrane.	Entire body (head, dorsum, and flanks) smooth, lacking prickles.	All fins yellow.
L. lunaris	Ventral half of body yellow.	Head, dorsum, and ventral surface covered with small prickles.	Lower caudal-fin lobe with a long white marginal stripe.
T. xanthopterus	Dorsum with 3–4 broad, arched, blue-black bars.	Head, dorsum, and venter with strong prickles; dorsal and ventral spinulation areas separated.	All fins yellow (most distinctive diagnostic feature).
T. ocellatus	Lateral side above pectoral fin with a blackish-green, saddle-shaped blotch margined with orange, extending dorsally across the back.	Head, dorsum, and venter with fine, weak prickles.	Dorsal-fin base with a large, round black spot edged with orange.
T. niphobles	Dorsolateral side scattered with numerous small milky-white spots; a large, flat black spot above the posterior end of the pectoral fin, connecting with the transverse dorsal band.	Head, dorsum, and venter with extremely minute prickles.	—
T. poecilonotus	Dorsum with 6 dark brown transverse bands.	Head, dorsum, and venter densely covered with small prickles; dorsal and ventral areas connected into a single patch.	Upper and lower margins of caudal fin dark brown.
T. bimaculatus	Dorsum with >10 paired arc-shaped blue-brown transverse bands; dark brown spots on anterior and posterior	Head, dorsum, and venter with strong prickles; dorsal and ventral spinulation	—

	bases of pectoral fin.	areas separated.	
T. oblongus	Dorsolateral side with >10 pairs of yellowish-brown transverse bands.	Head, dorsum, and venter with strong prickles; dorsal and ventral spinulation areas connected anterior and posterior to the pectoral fins.	Lower lateral side, lips, and all fins yellow.
