# Supplementary material for: Distribution of Tetrodotoxin in Wild Pufferfish from the Southeastern Coastal Regions of China
Source: Toxins (Basel). 2026 Oct 20;18(10):458. doi: 10.3390/toxins18100458 (PMC13709651; doi:10.3390/toxins18100458)
Supplement: Supplementary file 1 [file toxins-18-00458-s001.zip › Table S3.pdf]

Table S3. Seasonal Statistical Analysis

Species	Tissue	Spring vs. Summer	Spring vs. Autumn	Spring vs. Winter	Summer vs. Autumn	Summer vs. Winter	Autumn vs. Winter
L. Wheeleri（female）	Eye	p<0.01	p<0.01	ns	ns	ns	ns
	Skin	p<0.01	p<0.01	ns	ns	p<0.05	p<0.05
	Muscle	p<0.01	p<0.01	ns	ns	ns	ns
	Qvary	p<0.01	p<0.01	ns	ns	ns	ns
	Liver	ns	p<0.01	ns	ns	ns	ns
	Digestive tract	p<0.01	p<0.01	ns	ns	ns	ns
	Kidneys	p<0.05	p<0.01	ns	ns	ns	ns
	Heart	ns	ns	ns	ns	ns	ns
L. Wheeleri（male）	Eye	p<0.05	p<0.01	ns	ns	ns	ns
	Skin	p<0.05	p<0.05	ns	ns	ns	ns
	Muscle	ns	ns	ns	ns	ns	ns
	Testicle	p<0.05	p<0.01	ns	ns	ns	ns
	Liver	ns	p<0.05	ns	ns	ns	ns
	Digestive tract	p<0.01	p<0.01	p<0.05	ns	ns	ns
	Kidneys	p<0.01	p<0.01	ns	ns	ns	ns
	Heart	ns	ns	ns	ns	ns	ns
L. Gloveri (female)	Eye	-	-	-	p<0.01	p<0.01	ns
	Skin	-	-	-	ns	ns	ns
	Muscle	-	-	-	ns	p<0.05	ns
	Qvary	-	-	-	p<0.05	ns	ns
	Liver	-	-	-	ns	ns	ns
	Digestive tract	-	-	-	ns	ns	ns
	Kidneys	-	-	-	ns	ns	ns
	Heart	-	-	-	p<0.05	p<0.05	ns
L. Gloveri (male)	Eye	-	-	-	ns	ns	ns
	Skin	-	-	-	ns	ns	ns
	Muscle	-	-	-	ns	ns	ns
	Testicle	-	-	-	ns	ns	ns
	Liver	-	-	-	p<0.01	p<0.05	ns
	Digestive tract	-	-	-	ns	ns	ns
	Kidneys	-	-	-	ns	ns	ns
	Heart	-	-	-	p<0.01	ns	ns

Species		Spring vs. Summer	Spring vs. Autumn	Spring vs. Winter	Summer vs. Autumn	Summer vs. Winter	Autumn vs. Winter
L. Inermis (female)	Eye	-	-	-	-	ns	-
	Skin	-	-	-	-	ns	-
	Muscle	-	-	-	-	ns	-
	Qvary	-	-	-	-	p<0.05	-
	Liver	-	-	-	-	ns	-
	Digestive tract	-	-	-	-	ns	-
	Kidneys	-	-	-	-	ns	-
	Heart	-	-	-	-	ns	-
L. Inermis (male)	Eye	-	-	-	p<0.01	ns	ns
	Skin	-	-	-	ns	ns	ns
	Muscle	-	-	-	ns	ns	ns
	Testicle	-	-	-	p<0.01	ns	ns
	Liver	-	-	-	ns	ns	ns
	Digestive tract	-	-	-	ns	p<0.05	ns
	Kidneys	-	-	-	ns	p<0.01	ns
	Heart	-	-	-	ns	p<0.01	ns
L. Lunaris (female)	Eye	-	-	-	-	ns	-
	Skin	-	-	-	-	p<0.05	-
	Muscle	-	-	-	-	ns	-
	Qvary	-	-	-	-	p<0.05	-
	Liver	-	-	-	-	p<0.05	-
	Digestive tract	-	-	-	-	ns	-
	Kidneys	-	-	-	-	ns	-
	Heart	-	-	-	-	ns	-
L. Lunaris (male)	Eye	-	-	-	-	ns	-
	Skin	-	-	-	-	ns	-
	Muscle	-	-	-	-	ns	-
	Testicle	-	-	-	-	ns	-
	Liver	-	-	-	-	ns	-
	Digestive tract	-	-	-	-	ns	-
	Kidneys	-	-	-	-	ns	-
	Heart	-	-	-	-	ns	-

Species		Spring vs. Summer	Spring vs. Autumn	Spring vs. Winter	Summer vs. Autu mn	Summer vs. Winter	Autumn vs. Winter
T. Xanthopterus (female)	Eye	-	-	-	-	-	*p*<0.05
	Skin	-	-	-	-	-	ns
	Muscle	-	-	-	-	-	*p*<0.05
	Qvary	-	-	-	-	-	*p*<0.05
	Liver	-	-	-	-	-	*p*<0.05
	Digestive tract	-	-	-	-	-	ns
	Kidneys	-	-	-	-	-	ns
	Heart	-	-	-	-	-	*p*<0.05
T. Xanthopterus (male)	Eye	-	-	-	-	-	ns
	Skin	-	-	-	-	-	ns
	Muscle	-	-	-	-	-	*p*<0.05
	Testicle	-	-	-	-	-	*p*<0.05
	Liver	-	-	-	-	-	*p*<0.05
	Digestive tract	-	-	-	-	-	*p*<0.05
	Kidneys	-	-	-	-	-	ns
	Heart	-	-	-	-	-	*p*<0.05
T. ocellatus (male)	Eye	-	-	-	-	ns	-
	Skin	-	-	-	-	ns	-
	Muscle	-	-	-	-	ns	-
	Testicle	-	-	-	-	ns	-
	Liver	-	-	-	-	ns	-
	Digestive tract	-	-	-	-	ns	-
	Kidneys	-	-	-	-	ns	-
	Heart	-	-	-	-	ns	-

Species		Spring vs. Summer	Spring vs. Autumn	Spring vs. Winter	Summer vs. Autumn	Summer vs. Winter	Autumn vs. Winter
T. Poecilonotus (female)	Eye	-	-	-	ns	ns	ns
	Skin	-	-	-	ns	ns	ns
	Muscle	-	-	-	ns	ns	ns
	Qvary	-	-	-	p<0.01	ns	p<0.05
	Liver	-	-	-	ns	ns	ns
	Digestive tract	-	-	-	ns	ns	ns
	Kidneys	-	-	-	ns	ns	ns
	Heart	-	-	-	ns	ns	ns
T. Poecilonotus (male)	Eye	-	-	-	ns	ns	ns
	Skin	-	-	-	ns	ns	ns
	Muscle	-	-	-	ns	ns	ns
	Testicle	-	-	-	p<0.01	p<0.01	ns
	Liver	-	-	-	ns	ns	ns
	Digestive tract	-	-	-	ns	ns	p<0.05
	Kidneys	-	-	-	ns	p<0.05	ns
	Heart	-	-	-	ns	ns	p<0.05
T. Oblongus (female)	Eye	-	-	-	ns	-	-
	Skin	-	-	-	ns	-	-
	Muscle	-	-	-	ns	-	-
	Qvary	-	-	-	p<0.01	-	-
	Liver	-	-	-	ns	-	-
	Digestive tract	-	-	-	ns	-	-
	Kidneys	-	-	-	ns	-	-
	Heart	-	-	-	ns	-	-
T. Oblongus (male)	Eye	-	-	-	ns	-	-
	Skin	-	-	-	ns	-	-
	Muscle	-	-	-	ns	-	-
	Testicle	-	-	-	p<0.01	-	-
	Liver	-	-	-	ns	-	-
	Digestive tract	-	-	-	ns	-	-
	Kidneys	-	-	-	ns	-	-
	Heart	-	-	-	ns	-	-

Note:"ns" indicates no significant difference between two seasons.
