# Supplementary material for: Distribution of Tetrodotoxin in Wild Pufferfish from the Southeastern Coastal Regions of China
Source: Toxins (Basel). 2026 Oct 20;18(10):458. doi: 10.3390/toxins18100458 (PMC13709651; doi:10.3390/toxins18100458)
Supplement: Supplementary file 1 [file toxins-18-00458-s001.zip › Table S4.pdf]

Table S4 Summary of sample information

Species	Province	Sampling site	Sampling period	Sample size (n)
L. wheeleri	Fujian	East China Sea; sea areas 302 and 304; Taiwan Strait	2020-03, 2020-07	77
	Guangdong	South China Sea; sea areas 303, 373, 374, 314, 401 and 333	2019-09, 2020-10	82
	Guangxi	Sea area 362, and 363; The southern coast of Beihai, Guangxi	2019-09, 2019-12, 2020-10	80
	Hainan	The southwest of Yinggehai; sea areas 492, 514, 249 and 250	2019-09, 2020-09, 2020-10	87
L. gloveri	Fujian	Taiwan Strait; sea area 303	2019-09, 2019-10	24
	Guangdong	Maoming sea area; sea areas 426, 427, 374, 314, 401 and 333	2019-11, 2019-12, 202-01, 2020-09, 2020-10	72
	Guangxi	Sea area 362, and 363; The southern coast of Beihai, Guangxi	2019-09	6
	Hainan	The southwest of Yinggehai; Qinglan Fishing Ground	2019-09, 2019-12, 2020-07, 2020-08	58
L. inermis	Fujian	Ningde Xiapu	2019-10, 2019-12	82
	Guangdong	Maoming sea area;	2020-08	21
	Hainan	Sea area 514	2020-07	3
L. lunaris	Hainan	Sea areas 294 and 514	2019-12, 2020-07	35
T. xanthopterus	Fujian	Sea area 304; Eastern Fujian	2019-10, 2019-12	35
	Guangdong	Sea area 371	2019-09, 2019-11	11
T. ocellatus	Fujian	Sea area 304; Eastern Fujian	2019-09, 2019-12	20
	Guangdong	Zhuhai sea area	2019-12	4
T. niphobles	Guangxi	Sea area 363	2020-07	130
T. poecilonotus	Fujian	Taiwan Strait; sea area 303	2019-09	3
	Guangdong	Maoming sea area	2019-12	9
	Guangxi	Sea area 363	2019-12	12
T. bimaculatus	Fujian	Taiwan Strait; sea area 304;	2020-08	22
T. oblongus	Fujian	Taiwan Strait; sea area 304; Eastern Fujian	2019-10	51
	Guangdong	Maoming sea area	2019-10, 2020-07	150
	Guangxi	Sea areas 363, 425 and 426	2020-07	48
	Hainan	The southwest of Yinggehai	2019-11	108
